# Genome Sequence of *Escherichia coli* O157:H7 Strain 2886-75, Associated with the First Reported Case of Human Infection in the United States

**DOI:** 10.1128/genomeA.01120-13

**Published:** 2014-01-09

**Authors:** Fatemeh Sanjar, Tracy H. Hazen, Sadiq M. Shah, Sara S. K. Koenig, Sonia Agrawal, Sean Daugherty, Lisa Sadzewicz, Luke J. Tallon, Mark K. Mammel, Peter Feng, Robert Soderlund, Phillip I. Tarr, Chitrita DebRoy, Edward G. Dudley, Thomas A. Cebula, Jacques Ravel, Claire M. Fraser, David A. Rasko, Mark Eppinger

**Affiliations:** University of Texas at San Antonio, Department of Biology and South Texas Center for Emerging Infectious Diseases, San Antonio, Texas, USAa; Institute for Genome Sciences (IGS), University of Maryland, School of Medicine, Department of Microbiology and Immunology, Baltimore, Maryland, USAb; Centers for Food Safety and Applied Nutrition, U.S. Food and Drug Administration, Laurel, Maryland, USAc; National Veterinary Institute, Department of Bacteriology, SVA, Uppsala, Swedend; Washington University School of Medicine, St. Louis, Missouri, USAe; Pennsylvania State University, Department of Veterinary and Biomedical Sciences, University Park, Pennsylvania, USAf; Pennsylvania State University, Department of Food Science, University Park, Pennsylvania, USAg; Johns Hopkins University, Department of Biology, Baltimore, Maryland, USAh

## Abstract

First identified in 1982 as a human pathogen, enterohemorrhagic *Escherichia coli* of the O157:H7 serotype is a major cause of food-borne acquired human infections. Here, we report the genome sequence of the first known strain of this serotype isolated in the United States.

## GENOME ANNOUNCEMENT

Since the initial report in 1982 that *Escherichia coli* O157:H7 is associated with severe human disease, the serotype O157:H7 has assumed a position of dominance among enterohemorrhagic *E*. *coli* (EHEC) serotypes in North America responsible for global widespread outbreaks of severe gastrointestinal disease (1, 2). This lineage of Shiga toxin-producing *E. coli* (STEC) O157:H7 is non-sorbitol fermenting and β-glucuronidase negative and has evolved from an O55:H7 progenitor (3, 4). The isolation in 1975 of this *E. coli* O157:H7 strain, designated 2886-75, from an adult with hemorrhagic colitis (HC) (5, 6) predated the 1982 Oregon and Michigan hamburger-associated *E. coli* O157:H7 outbreaks (6). Since 1982, this serogroup has emerged as the dominant cause of EHEC infections in North America. Infections typically present with symptoms of bloody diarrhea coupled with severe abdominal pain (5, 6) but can rapidly progress to life-threatening complications, such as hemolytic uremic syndrome (HUS), HC, and central nervous system failure (7–12).

Genomic DNA was subjected to Illumina sequencing using paired-end libraries with 300-bp inserts on the HiSeq platform. The draft genome was assembled with Velvet assembler (13, 14), and the IGS Annotation Engine and Manatee were used for genome annotation and visualization (15). Availability of the high-quality genome sequence enabled the determination of the pathogenome virulence state (16) and phylogenomic grouping according to established genotypic classification methods using *in-silico* and experimental assays (17–20). PCR genotyping confirmed the *stx* genotype and determined the occupancy of both the *yehV* and *wrbA* bacteriophage insertion sites (21). Strain 2886-75 has an unusual genotype. Unlike the majority of *E. coli* O157:H7 recovered from humans in the United States (22–24), this isolate is *stx*_1_ positive and *stx*_2_ negative. The *yehV* site is occupied by the *stx*_1_ bacteriophage that is not stably integrated. Hence, the genomic architecture does not fit the emergence scenario typical of other human-pathogenic *E. coli* O157:H7 strains, and this isolate cannot be placed into clusters 1, 2, or 3 (25). However, this strain shows other typical genetic hallmarks of EHEC. Strain 2886-75 carries the lineage-specific virulence plasmid pO157 (26, 27), the T allele of the translocated intimin receptor (*tir*) (255 T>A), and a chimeric polymorphic variant of repeat region 1 (RR1) with the absence of the repeat regions RR2 and RR3, placing strain 2886-75 closest to group 8 (28). Multilocus sequence typing (MLST) (18) based on the nucleotide sequences of 15 housekeeping genes revealed that 2886-75 exhibits allele combination 23.11 (19) and belongs to the sequence type 11 (ST11) and complex/ABD group (18, 20). Strain 2886-75 is a representative of lineage I (17, 29) and clade 3.16 (30). The genome sequence presented here will be a valuable resource in studying *E. coli* O157:H7 pathogenome evolution by comparing this isolate to the extant genotypes and will aid in the development of a higher-resolution phylogenomic framework for improved molecular-guided pathogen surveillance and outbreak investigations (10, 11, 31).

### Nucleotide sequence accession number.

This genome sequence is deposited in GenBank under the accession number AVRR00000000. A bacterial strain culture is available from the Biodefense and Emerging Infections Research Resources Repository (http://www.beiresources.org/).
